# Enhancing Performance of Thin-Film Nanocomposite Membranes by Embedding in Situ Silica Nanoparticles

**DOI:** 10.3390/membranes12060607

**Published:** 2022-06-11

**Authors:** Manuel Reyes De Guzman, Micah Belle Marie Yap Ang, Kai-Ting Hsu, Min-Yi Chu, Jeremiah C. Millare, Shu-Hsien Huang, Hui-An Tsai, Kueir-Rarn Lee

**Affiliations:** 1 Material Corrosion and Protection Key Laboratory of Sichuan Province, School of Materials Science and Engineering, Sichuan University of Science and Engineering, Zigong 643000, China; manuelrdg@yahoo.com; 2 R&D Center for Membrane Technology, Department of Chemical Engineering, Chung Yuan Christian University, Taoyuan 32023, Taiwan; mbmyang@gmail.com (M.B.M.Y.A.); chuminyi1029@gmail.com (M.-Y.C.); huian@cycu.edu.tw (H.-A.T.); krlee@cycu.edu.tw (K.-R.L.); 3 Department of Chemical and Materials Engineering, National Ilan University, Yilan 26047, Taiwan; candy7799@hotmail.com; 4 School of Chemical, Biological, and Materials Engineering and Sciences, Mapúa University, Manila 1002, Philippines; jcmillare@mapua.edu.ph; 5 Research Center for Circular Economy, Chung Yuan Christian University, Taoyuan 32023, Taiwan

**Keywords:** thin-film, membrane, silica, pervaporation, interfacial polymerization

## Abstract

In this work, silica nanoparticles were produced in situ, to be embedded eventually in the polyamide layer formed during interfacial polymerization for fabricating thin-film nanocomposite membranes with enhanced performance for dehydrating isopropanol solution. The nanoparticles were synthesized through a sol-gel reaction between 3-aminopropyltrimethoxysilane (APTMOS) and 1,3-cyclohexanediamine (CHDA). Two monomers—CHDA (with APTMOS) and trimesoyl chloride—were reacted on a hydrolyzed polyacrylonitrile (hPAN) support. To obtain optimum fabricating conditions, the ratio of APTMOS to CHDA and reaction time were varied. Field emission scanning electron microscopy (FESEM) and atomic force microscopy (AFM) were used to illustrate the change in morphology as a result of embedding silica nanoparticles. The optimal conditions for preparing the nanocomposite membrane turned out to be 0.15 (g/g) APTMOS/CHDA and 60 min mixing of APTMOS and CHDA, leading to the following membrane performance: flux = 1071 ± 79 g∙m^−2^∙h^−1^, water concentration in permeate = 97.34 ± 0.61%, and separation factor = 85.39. A stable performance was shown by the membrane under different operating conditions, where the water concentration in permeate was more than 90 wt%. Therefore, the embedment of silica nanoparticles generated in situ enhanced the separation efficiency of the membrane.

## 1. Introduction

Membrane technology replaces traditional separation processes because it is considered to be greener and more economical. Fabricating superior membranes is one of the key factors in obtaining high separation efficiencies. Different fabrication techniques have been applied to produce a wide variety of porous or dense membranes. Porous membranes are suitable for microfiltration, ultrafiltration, and membrane distillation, whereas dense membranes are usually used for reverse osmosis, forward osmosis, gas separation, pervaporation, and vapor permeation [1,2]. For solvent dehydration, pervaporation is the most commonly used separation process. Solvents, such as alcohols, tetrahydrofuran, and acetic acid, have been dehydrated through pervaporation [3].

Polymeric membranes are widely used for pervaporation dehydration because they are cheap, flexible, easy to process, and cost-effective. Membranes prepared through interfacial polymerization demonstrate a promising performance compared to those prepared through a dry-phase inversion process. Such membranes are called thin-film composite (TFC) membranes, in which a reaction occurs between an aqueous-phase monomer and an organic-phase monomer [4]. TFC membranes are usually composed of a dense selective layer (10–200 nm) on top of a porous support, and this layer is responsible for high separation efficiencies. Furthermore, each layer can be optimized to produce membranes with a higher performance [5]. Depending on the solvent, however, polymeric membranes are prone to swelling; thus, many researchers modify polymeric membranes with inorganic materials, such as silica [6,7,8,9,10], titanium dioxide [11,12,13,14], silver [15,16,17], zeolites [18,19,20,21,22], molybdenum sulfide [23,24,25], and clay [26,27,28,29].

Among the inorganic materials, silica is the most widely used because its synthesis is low-cost and easy to control. In preparing TFC membranes, inorganic particles can be mixed with the aqueous phase or the organic phase to embed them in the resultant thin selective layer. The process of synthesizing inorganic particles is an additional step considered by several studies in fabricating membranes with enhanced performance. For example, Fathizadeh et al. [22] dispersed nano-NaX zeolite in an organic phase of trimesoyl chloride (TMC) to fabricate thin-film nanocomposite (TFN) membranes. They found that, at an appropriate concentration of nano-NaX zeolite, the permeation flux and separation factor were enhanced. Vatanpour et al. [14] dispersed titanium oxide/carbon dots in the aqueous phase to improve the membrane performance. Their modified membrane exhibited a smoother surface and a lower water contact angle than the unmodified one; in turn, the TFN polyamide membrane had better permeability. Layered double hydroxides (LDH), a type of clay, was mixed with an organic TMC solution by Zhao et al. [26], and it was consequently embedded in the polyamide layer formed at the end of the interfacial polymerization process. The water permeability of their membrane improved without sacrificing its selectivity because of the moderate enlargement of the interlayer spacing in LDH. Cheng et al. [30] dispersed graphene oxide in an aqueous phase to fabricate a nanofibrous TFC membrane. The embedment of graphene oxide was conducive to delivering a high permeation flux, along with an improved separation factor, during the dehydration of isopropanol at 70 °C. Ang et al. [9,10] synthesized different sizes of silica nanospheres and different shapes of hollow silica. Suitable size and shape improved both the water flux and the antifouling property. All the aforementioned studies synthesized the nanoparticles separately. Therefore, the cost of the production of membranes was more expensive because of the additional synthesis processes and purification steps.

The innovation in this work is that silica nanoparticles were synthesized in situ. That is, the production of the nanoparticles was on-site. They were formed from the sol-gel reaction between 3-aminopropyltrimethoxysilane (APTMOS) and 1,3-cyclohexanediamine (CHDA); the ratio of APTMOS to CHDA was varied. Thus, no separate steps for the separation and purification of nanoparticles were necessary, and these are the advantages of our approach over that adopted by the previous studies described above. The growth of the silica nanoparticles was affected by the APTMOS concentration and the duration of stirring during the synthesis. This aqueous solution of CHDA with in situ-generated silica nanoparticles was directly used to react with TMC to carry out interfacial polymerization. As a result, a polyamide layer with embedded silica nanoparticles was formed on a hPAN support. The embedment of silica nanoparticles generated in situ improved the membrane selectivity. Through this simplified approach, which integrated in situ formation of nanoparticles (i.e., the synthesis occurred in place), the cost of fabricating nanocomposite membranes would be reduced.

## 2. Materials and Methods

### 2.1. Materials

Polyacrylonitrile (PAN) was provided by Tong-Hwa Synthesis Fiber Co. Ltd. (Taipei, Taiwan). N-methyl-2-pyrrolidone (NMP) was supplied by Tedia Company Inc. (Fairfield, OH, USA). CHDA and TMC were bought from Tokyo Chemical Industry Co. Ltd. (Tokyo, Japan). APTMOS was manufactured by Sigma-Aldrich (Saint Louis, MO, USA). Toluene, and methanol were acquired from Echo Chemical (Miaoli, Taiwan). IPA was provided by UNI-ONWARD Corp., New Taipei City, Taiwan. Sodium hydroxide was from Fullin Chemical Co. Ltd., Taipei, Taiwan.

### 2.2. Synthesis of Hydrolyzed Polyacrylonitrile Support

In a 100 mL bottle, 15 g of PAN was dissolved in 85 mL NMP at room temperature, with constant stirring at 200 rpm for 24 h. The solution was degassed for 1 day to remove the bubbles generated from the previous step. Afterward, it was cast on nonwoven polyester using a casting knife with a gap of 100 µm. The cast film was immediately immersed in water for solidification, forming the PAN support. It was washed with water several times to completely remove the excess NMP. For the process of hydrolysis, the wet PAN support was immersed in 2 M NaOH at 50 °C for 10 min. Finally, the hPAN support was washed until the pH of the wash solution became neutral. Prior to interfacial polymerization, the hPAN was stored in distilled water.

### 2.3. Fabrication of Silica-Modified Thin-Film Nanocomposite Membranes

**Figure 1:**
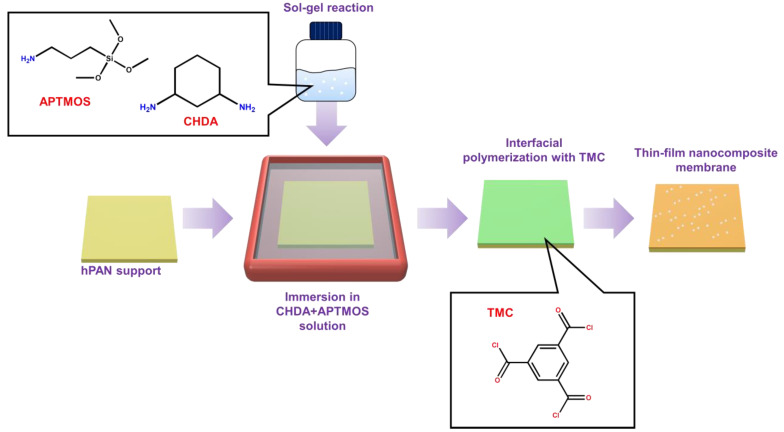
Schematic diagram of preparing a thin-film nanocomposite membrane with embedded silica nanoparticles generated in situ from the reaction between APTMOS and CHDA.

Figure 1 illustrates the membrane fabrication. A 0.5 wt% TMC/toluene solution was prepared as an organic-phase solution. CHDA and different amounts of APTMOS were dissolved in water, where the concentration of CHDA was fixed at 0.5 wt%. The solution of CHDA with APTMOS was stirred for 1 h to carry out the sol-gel reaction. After that, the hPAN was immersed in the aqueous-phase solution for 5 min. The excess solution was then gently pressed out using a glass rod. Subsequently, the TMC solution was poured onto the hPAN support to induce the interfacial polymerization reaction between CHDA and TMC for 3 min. Finally, the membrane was dried at room temperature. To remove the excess monomers, it was washed with methanol and dried again at room temperature.

### 2.4. Membrane Characterization

The surface chemical functional groups and elemental composition were examined using attenuated total reflectance–Fourier transform infrared (ATR-FTIR) spectroscopy (Perkin Elmer Spectrum 100 FTIR Spectrometer, Waltham, MA, USA) and K-Alpha™ + X-ray photoelectron spectrometry (XPS, ThermoFisher Scientific Inc., Renfrew, UK), respectively. The membrane was vacuum-dried before it was placed on the ATR-FTIR sample stage. Field emission scanning electron microscopy (FESEM, S-4800, Hitachi Co, Tokyo, Japan) was used to capture the surface and cross-sectional images of the membranes. For the determination of the surface morphology, samples were attached to an FESEM stage with carbon tape, and for the cross-sectional FESEM images, samples were freeze-fractured, and then the fractured samples were affixed to the FESEM stage. Before the FESEM test, the samples were sputtered with Pt dust to protect the membranes during the test. Nanoparticle sizes were analyzed using transmission electron microscopy (TEM, JEOL JEM-2100, Tokyo, Japan). To measure the thickness of the selective layer and the size of the nanoparticles, ImageJ software was used. Surface roughness (root mean square, Rq) was quantified using atomic force microscopy (AFM, NanoScope^®^ V, Bruker, Billerica, MA, USA). Samples were attached to an AFM sample stage. The image was then captured with a scan size of 10 × 10 µm. The membrane hydrophilicity was evaluated using an automatic interfacial tensiometer (PD-VP Model, Kyowa Interface Science Co. Ltd., Niiza-City, Saitama, Japan). After 1 min of contact between the water drop and the membrane surface, the water contact angle was measured and recorded.

### 2.5. Pervaporation Test

The membranes, having an effective area (A) of 7.07 cm^2^, were tested using a laboratory-made pervaporation apparatus [31]. A 70% aqueous isopropyl alcohol (IPA) solution at 25 °C was fed into the system. The downstream pressure was fixed at 1.0 cmHg, and the sampling time was after 15 min. A trap was employed to collect the permeate, which was frozen at the point of collection, as the trap was immersed in liquid nitrogen. Gas chromatography (China Chromatography, GC 2000, Taipei, Taiwan) was used to measure the concentrations of feed and permeate. The permeation flux (*J*) and separation factor (*β*) were calculated using the following equations.

(1) $$J=\frac{m}{At}$$

(2) $$\beta =\frac{Y_{w}/Y_{IPA}}{X_{W}/\;X_{IPA}}$$

The mass in the trap was represented by *m*, which was collected after 15 min of sampling time (*t*). *Y_W_* and *X_W_* were the concentrations of water in permeate and feed, respectively. *Y_IPA_* and *X_IPA_* were the concentrations of IPA in permeate and feed, respectively.

## 3. Results and Discussion

### 3.1. Surface Chemical Property

**Figure 2:**
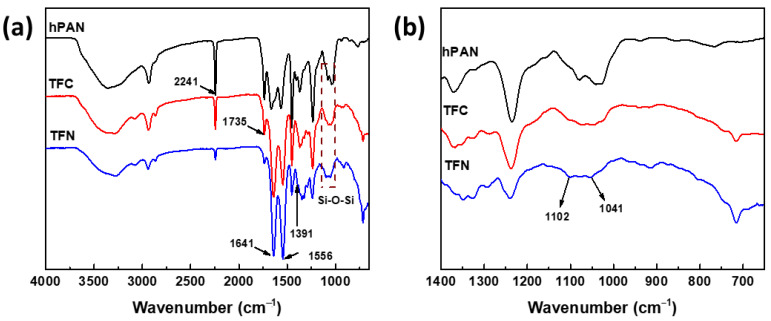
ATR-FTIR spectra of hPAN, TFC, and TFN membranes: (**a**) wavenumber from 4000 to 650 cm^−1^; (**b**) wavenumber from 1400 to 650 cm^−1^.

Figure 2 indicates the ATR-FTIR spectra of three membranes. The peak at 2241 cm^−1^ of hPAN corresponds with stretching vibration of CN bands, whereas the peak at 1735 cm^−1^ is attributed to CO stretching bands. As a result of interfacial polymerization between CHDA (with APTMOS) and TMC, a polyamide layer was formed on top of the hPAN support, and its formation is evidenced by the appearance of strong peaks of amide I and amide II at 1641 and 1556 cm^−1^, respectively [32]. The peak at 1391 cm^−1^ of TFN is attributed to CH_2_ vibration of APTMOS, and changes in the peak around 1102–1041 cm^−1^ (Figure 2b) suggest the embedment of silica coming from the Si-O-Si chain [33]. The chemical compositions of TFC and TFN membranes, as deduced from their XPS analysis, are listed in Table 1. TFN has 1.29% Si on its surface, implying that APTMOS underwent a sol-gel reaction to form a network with CHDA. Therefore, silica nanoparticles were generated in situ during the preparation of TFN through interfacial polymerization.

### 3.2. Morphology and Water Contact Angle Analysis

**Figure 3:**
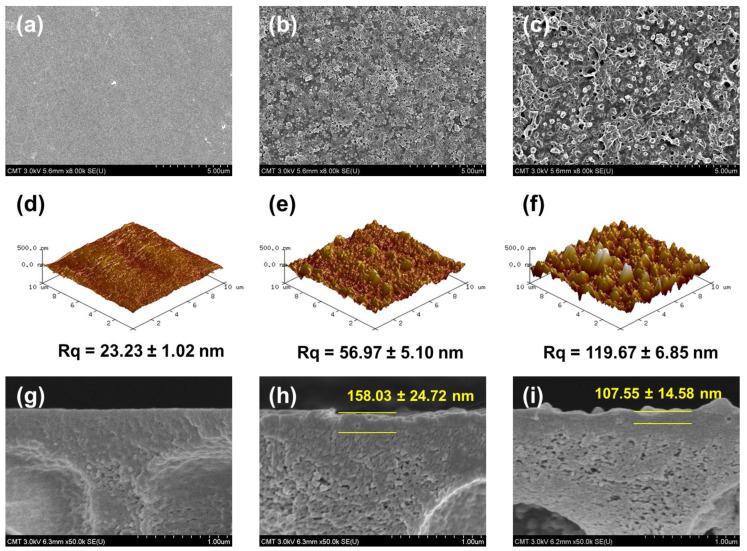
FESEM and AFM morphology: (**a**,**d**,**g**) hPAN; (**b**,**e**,**h**) TFC; and (**c**,**f**,**i**) TFN membranes.

The surface of the hPAN membrane is smooth (Figure 3a). Among the three membranes described in Figure 3, hPAN has the lowest surface roughness (Rq = 23.23 ± 1.02 nm) (Figure 3d). From the interfacial polymerization between CHDA and TMC on top of hPAN, a TFC membrane was formed. Its structure shows nodules on the surface (Figure 3b), the roughness of which is 56.97 ± 5.10 nm (Figure 3e). With the incorporation of APTMOS into CHDA, silica nanoparticles were generated in situ by way of a sol-gel reaction, and the surface of the resulting membrane is shown to be very rough (Figure 3c), indicated by a surface roughness of 119.67 ± 6.85 nm (Figure 3f). Distinct silica nanoparticles can also be observed (Figure 3c).

**Figure 4:**
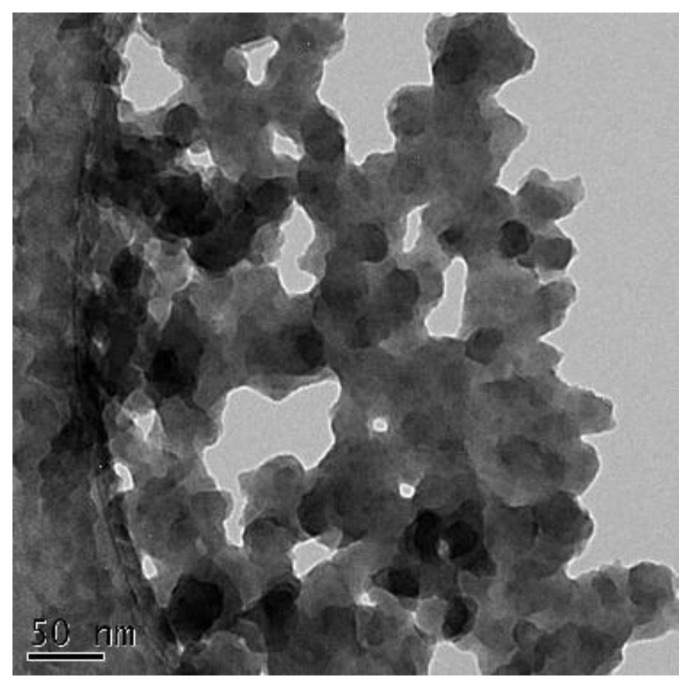
TEM image of silica nanoparticles embedded in the polyamide layer.

To determine the size of the silica nanoparticles embedded in the polyamide layer, the following procedure was conducted. A solution of CHDA with APTMOS was dropped onto a copper mesh (200-mesh size) coated with a carbon film, which served as a substrate. A drop of TMC solution was then added to the CHDA solution. Upon contact of the two solutions, interfacial polymerization immediately took place. A polyamide layer with embedded silica nanoparticles was formed on the substrate. Afterward, the unreacted monomers were washed away with methanol, and the substrate with a layer of polyamide-silica was vacuum-dried. Through TEM, the nanoparticle size was measured to be 27.45 ± 5.87 nm (Figure 4). The TEM image provides support that silica nanoparticles were formed from the reaction between APTMOS and CHDA, which were eventually embedded in the polyamide layer.

It can be discerned from Figure 3h,i that the TFC membrane has a thicker polyamide layer (158.03 ± 24.72 nm) than the TFN membrane (107.55 ± 14.58 nm). The presence of silica nanoparticles in the CHDA solution hindered the reaction between CHDA and TMC, resulting in a thinner polyamide layer for the TFN membrane. Furthermore, amine in APTMOS could also react with TMC, and this is also a probable reason for the production of a thinner polyamide layer.

**Figure 5:**
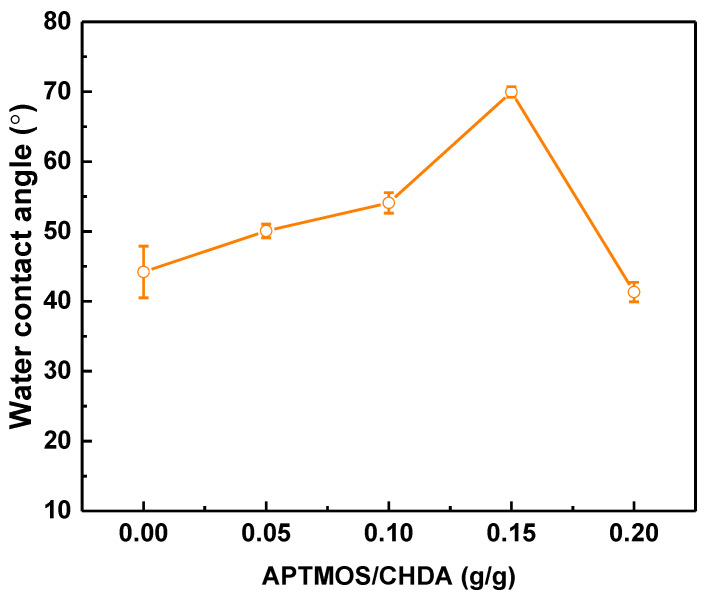
Water contact angles of various TFN membranes, depending on the ratio of APTMOS to CHDA.

Figure 5 presents the water contact angle of different TFN membranes. Increasing the ratio of APTMOS to CHDA from 0 to 0.15 also increases the contact angle from 44.2 ± 3.7° to 69.95 ± 0.77°. This increase is because of the presence of CH_2_ groups in silica nanoparticles on the membrane surface, making the membrane less hydrophilic. This is the effect of the presence of silica nanoparticles; having a less hydrophilic surface adversely affects the membrane performance. The next section discusses the advantages of embedding silica nanoparticles in the polyamide layer.

### 3.3. Membrane Performance

**Figure 6:**
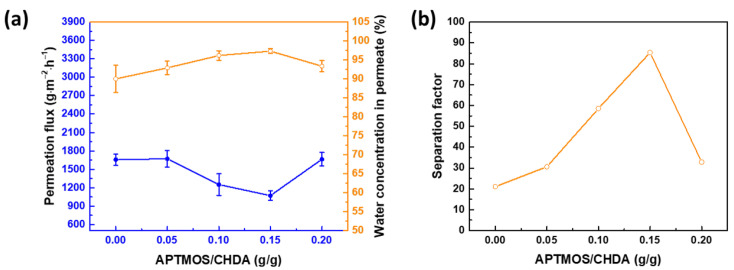
Effect of the APTMOS/CHDA ratio on pervaporation membrane performance, as a function of (**a**) permeation flux and water concentration in permeate and (**b**) separation factor. Time for mixing CHDA and APTMOS = 60 min; reaction temperature = 30 °C; concentration of IPA = 70 wt%; downstream pressure = 1 mmHg; and operating temperature = 25 °C.

The conditions for fabricating the membranes were varied in terms of the following variables: ratio of APTMOS to CHDA and mixing time. The optimum conditions would give the highest membrane performance. Figure 6a illustrates the effect of the ratio of APTMOS to CHDA on separation efficiency. From 0 to 0.15 g APTMOS/g CHDA, the flux decreases from 1659 ± 90 to 1071 ± 79 g∙m^−2^∙h^−1^, whereas the water concentration in permeate increases from 90.02 ± 3.62% (β = 21.05, taken from Figure 6b) to 97.34 ± 0.61% (β = 85.39). Adding APTMOS created a new Si-O-Si network around the polyamide chain, leading to the blockage of defects in the chain, resulting in high selectivity. However, increasing the ratio to 0.2 g APTMOS/g CHDA led to oversaturation of silica nanoparticles or too much silica network in the aqueous phase, causing an interruption in the reaction between CHDA and TMC. This interruption created more defects in the selective layer, resulting in low efficiency when separating water from isopropanol. Therefore, the optimal ratio is 0.15 g/g APTMOS/CHDA.

**Figure 7:**
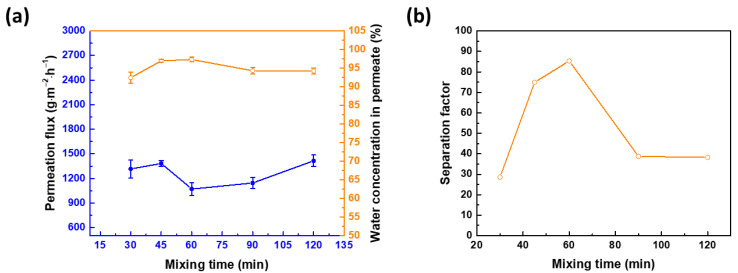
Effect of APTMOS and CHDA mixing time on pervaporation membrane performance, as a function of (**a**) permeation flux and water concentration in permeate and (**b**) separation factor. APTMOS/CHDA ratio = 0.15; reaction temperature = 30 °C; concentration of IPA = 70 wt%; downstream pressure = 1 mmHg; and operating temperature = 25 °C.

The time for mixing CHDA and APTMOS affects the growth of silica nanoparticles in an aqueous CHDA solution (Figure 7). From 30 to 60 min, the permeation flux decreases while the water concentration in permeate increases (Figure 7a). When the mixing time is less than 60 min, the amount of silica nanoparticles in an aqueous CHDA solution is probably inadequate to cover up the defects in the polyamide network, and this results in an increased separation factor (Figure 7b). However, when the mixing time is 120 min, the permeation flux increases to 1416 ± 70 g∙m^−2^∙h^−1^, with the water concentration in permeate equal to 94.25 ± 0.83% (β = 38.25, as deduced from Figure 7b). The low separation factor for a mixing time of more than 60 min is attributed to a lot of nanoparticles in an aqueous CHDA solution, leading to an aggregation of the nanoparticles and a strong interruption in the reaction between CHDA and TMC. This could result in a defective polyamide layer. Therefore, 60 min is the optimal mixing time.

### 3.4. Operating Conditions

**Figure 8:**
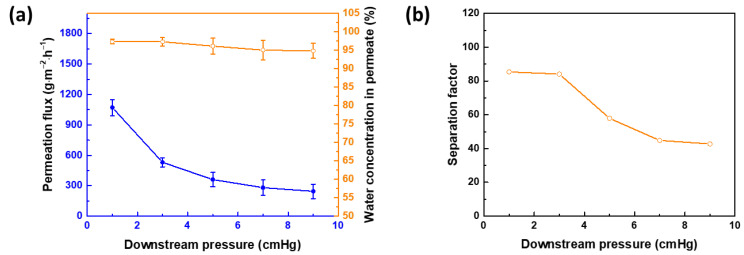
Membrane performance at different downstream pressures, in terms of (**a**) permeation flux and water concentration in permeate and (**b**) separation factor. APTMOS/CHDA ratio = 0.15; mixing time = 60 min; reaction temperature = 30 °C; concentration of IPA = 70 wt%; and operating temperature = 25 °C.

The operating conditions can be adjusted to attain the maximum membrane performance. Increasing the downstream pressure from 1 to 9 cmHg decreases both the permeation flux and the separation factor (Figure 8). The upstream pressure is constant at 760 mm Hg. At a low downstream pressure, the desorption rate of molecules during pervaporation is fast because of the strong driving force, leading to a high permeation flux. However, increasing the downstream pressure decreases the flux. According to Dalton’s law, the effect of augmenting the downstream pressure at the same upstream pressure is to weaken the vacuum (in other words, the driving force is reduced).

**Figure 9:**
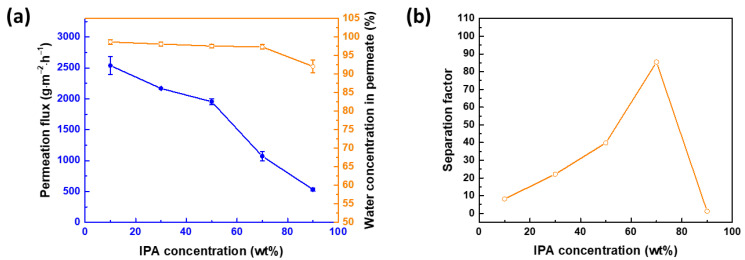
Membrane performance at different IPA concentrations in feed, in terms of (**a**) permeation flux and water concentration in permeate and (**b**) separation factor. APTMOS/CHDA ratio = 0.15; mixing time = 60 min; reaction temperature = 30 °C; downstream pressure = 1 mmHg; and operating temperature = 25 °C.

Figure 9a displays the membrane performance at different concentrations of IPA in the feed. From 10 to 90 wt% IPA, the permeation flux decreases from 2538 ± 143 to 531 ± 26 g∙m^−2^∙h^−1^. The water concentration in permeate also decreases from 98.66 ± 0.65 to 92.04 ± 1.71%. At a low concentration of IPA, there is a high driving force for water to penetrate the membrane, leading to a high permeation flux. As to the separation factor, it is optimal at 70 wt% IPA (Figure 9b).

**Figure 10:**
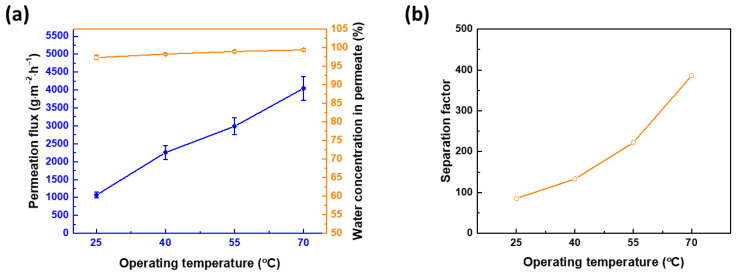
Membrane performance at different operating temperatures, in terms of (**a**) permeation flux and water concentration in permeate and (**b**) separation factor. APTMOS/CHDA ratio = 0.15; mixing time = 60 min; reaction temperature = 30 °C; concentration of IPA = 70 wt%; and downstream pressure = 1 mmHg.

Figure 10 evaluates the stability of TFN membranes at different temperatures. As can be discerned from Figure 10a, when the feed temperature increases from 25 to 70 °C, the permeation flux also increases from 1071 ± 79 to 4048 ± 330 g∙m^−2^∙h^−1^, while the water concentration in permeate also increases from 97.34 ± 0.61 to 99.4 ±0.3% (the equivalent separation factor is indicated in Figure 10b, increasing from β = 85.38 to β = 386). Increasing the temperature enhances the driving force, the effect of which is to hasten the motion of the molecules, resulting in an increased driving force of saturation vapor pressure on the upstream side. Thus, the permeation flux increases [34]. In addition, at a high temperature, the free volume in the membrane is enlarged, allowing more molecules to pass through easily. During pervaporation at high temperatures, the water molecules have a strong interaction with the membrane (for example, hydrogen bonding between the membrane and water), resulting in decreased dissolution or sorption rate. Therefore, the water molecules can be transported faster than IPA, leading to the enhancement of the separation factor at high temperatures.

**Figure 11:**
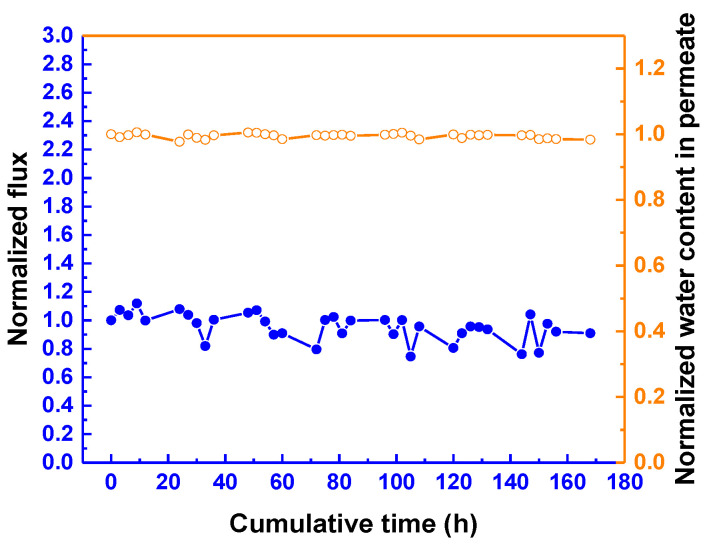
Membrane performance for a period of 168 h. APTMOS/CHDA ratio = 0.15; mixing time = 60 min; reaction temperature = 30 °C; concentration of IPA = 70 wt%; downstream pressure = 1 mmHg; and operating temperature = 25 °C.

Figure 11 demonstrates the long-term performance of the TFN membrane in circulated mode. For a period of 168 h, the membrane has a stable permeation flux and water concentration in permeate. These data suggest that the membrane appears to have no tendency to undergo swelling, even for a long period of operation. Table 2 lists the performance of the membrane, in comparison with that of several membranes reported in the literature. At a similar IPA concentration in feed and operating temperature, the overall performance of our TFN membrane is comparable to that of the other membranes. Although some membranes were reported to have a high separation factor, the permeation flux turned out be very low.

## 4. Conclusions

In this work, we adopted a simplified but versatile approach of fabricating TFN membranes with enhanced performance through the integration of silica nanoparticles generated in situ. The size of the embedded silica nanoparticles in the polyamide layer was 27.45 ± 5.87 nm. This embedment led to an increased water contact angle for the membrane, resulting in a higher separation factor. If the weight ratio of APTMOS to CHDA was 0.15 and the time of mixing APTMOS and CHDA was 60 min, then the resultant TFN membrane delivered optimum performance (flux = 1071 ± 79 g∙m^−2^∙h^−1^, water concentration in permeate = 97.34 ± 0.61%). Generating silica nanoparticles from the reaction between APTMOS and CHDA created a new Si-O-Si network around the resultant polyamide chain, which served to block the defects in the chain, and this translated to high selectivity. Embedding silica nanoparticles that were generated in situ enhanced the stability of TFN membranes subjected to different operating conditions, such as temperature, concentration of IPA in feed, and downstream pressure. The technique applied in this study is a step-up as far as incorporating inorganic nanoparticles into TFN membranes is concerned, as the need to separately synthesize, recover, and purify the nanoparticles is eliminated.

## Figures and Tables

**Table 1:** Elemental compositions of TFC and TFN membranes.

	C (%)	O (%)	N (%)	Si (%)
TFC	72.60	16.38	11.02	-
TFN	74.55	14.98	9.18	1.29

**Table 2:** Comparison of data on performance of various pervaporation membranes for dehydrating isopropanol solution.

Membrane	IPA in Feed (wt%)	Temperature (°C)	Permeation Flux (g∙m^−2^∙h^−1^)	Water Conc. in Permeate (wt%)	Separation Factor (β)	Reference
TFN	70	25	1071	97.34	85	This work
Chitosan-HMDI/PSf	70	30	1600	97.1	78	[35]
PDAA/PVDF	70	25	2411	95.7	52	[36]
HEC/SA/PAN	70	22	1212	95.54	50	[37]
CS/PSf	70	50	900	98	114	[38]
PVA-MA-PL (3 wt%)/PA-17	80	22	296	98.2	218	[39]
PVA-g-PNHMA	87.4	40	11	93.2	95	[40]
PVA-g-PNHMA	87.4	30	8.5	98.12	362	[41]
PERVAP^®^ 2201	70	60	300–400	98.5	153	[42]
PERVAP^®^ 2510	87.5	70	1100	99.2	868	[43]

## Data Availability

Not applicable.
